# Vulvar Buschke-Löwenstein tumor in a HPV 16 infected woman: case report

**DOI:** 10.11604/pamj.2022.42.50.35120

**Published:** 2022-05-18

**Authors:** Safia Ernez, Mouna Derouich, Salma Chachia, Sassi Boughizane

**Affiliations:** 1 Department of Gynecology and Obstetrics, University Hospital Farhat Hached, Street Doctor Moreau, 4000 Sousse, Tunisia

**Keywords:** Buschke Löwenstein tumor, human papillomavirus, verrucous lesion, case report

## Abstract

The Buschke-Löwenstein tumor is characterized by an exophytic lesion on the perianal region. It is considered benign but there is a high risk of recurrence and degenerative potential. It is commonly associated with human papillomavirus (HPV) especially subtypes 6 and 11, its evolution depends on the host's immunity and the association with other sexually transmitted diseases. Surgical excision is the recommended treatment in most cases. We report the case of a 54-year-old woman with only diabetes history, who had verrucous vulvar lesion associated to HPV subtype 16 treated with large excision.

## Introduction

Buschke-Löwenstein tumor is a rare exophytic benign lesion, mainly affecting the ano-genital areas. It is a sexually-transmitted disease linked to the human papillomavirus, especially type 6 and 11 [1]. This tumor can reach high dimension depending on the immune status and has a high degenerative potential and risk of recurrence [2]. The main treatment is surgical by large excision [3].

## Patient and observation

**Patient information**: we show the case of a 54-year-old patient with only diabetes history, who was referred to our consultation by her dermatologist after discovering an exophytic lesion involving the vulva.

**Figure 1:**
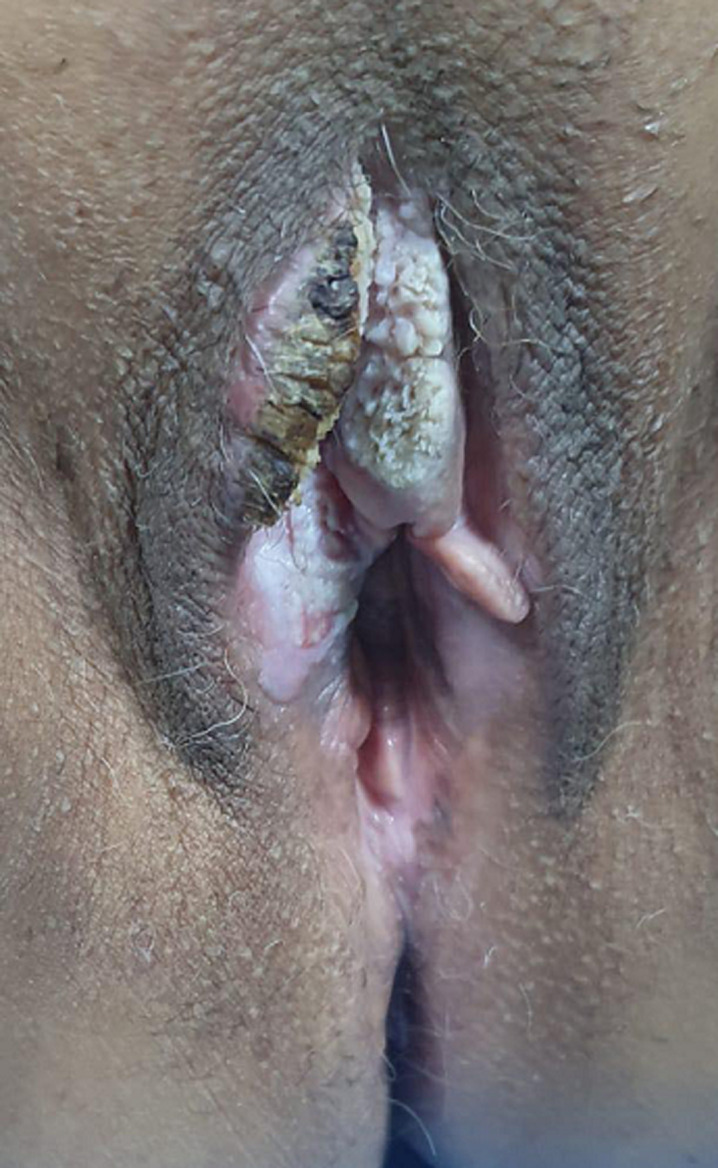
exophytic vulvar lesion suggestive of Buschke-Löwenstein tumor

**Clinical findings**: her physical examination revealed an exophytic cauliflower-like lesion involving the clitoris and 3/4 of the right hemi vulva (Figure 1), the rest of the examination was normal.

**Timeline of current episode**: the history began 6 months ago, with vulvar pruritus, and then after, a vulvar lesion appeared since few weeks.

**Diagnostic assessment**: local biopsy of the tumor was done.

**Diagnosis**: the histological examination of the biopsy from the tumor revealed a Buschke-Löwenstein tumor.

**Therapeutic interventions**: a large surgical excision of the tumor was done.

**Follow-up and outcome of interventions**: the operation was successful with no complication. HPV genotype 16 was detected on this tumor. The serologic screening for HIV was negative.

**Informed consent**: written informed consent was obtained from the patient.

## Discussion

Buschke-Löwenstein tumor is associated with HPV infection subtypes 6 and 11, which are highly contagious and are transmitted primarily through anogenital and oral sexual contact. This is frequently attributed to both early initiation of sexual intercourse and a high number of sexual partners. There is spontaneous regression in most of the time, however, in a small percentage of cases there is long-term persistence of warts, it depends on cofactors, such as host immunosuppression, patient age, and infection with oncogenic HPV genotypes, particularly 16 and 18 [4], as in our case the patient was 54, had diabetes which is a factor of immunosuppression and the coinfection of HPV subtype 16. The malignant transformation of Buschke-Löwenstein tumor depends on both of the action of HPV and the presence of other oncogenic factors. The infecting subtype of HPV is of great importance. Various reports in the literature suggest that a coinfection with high-risk HPV subtypes, mainly 16 and 18, is necessary for this transformation to occur [5].

In general, the malignant transformation process of this giant wart leads to the development of verrucous carcinoma, which is a well-differentiated, low-grade variant of squamous cell carcinoma [6]. Clinical treatment in Buschke-Löwenstein tumor should be preceded by a thorough clinical and pathological analysis to determine the extent of the lesion and the degree of tumor invasion [7]. Different treatment modalities have been used in individual cases. Many authors suggest that surgical excision of the tumor with a wide free margin, with or without adjuvant chemotherapy, is the gold standard of treatment for Buschke-Löwenstein tumor [7-9]. But there is a high number of local recurrence rate (66%) with any treatment modality [7] , which need a close surveillance.

## Conclusion

Buschke-Löwenstein tumor is a rare sexually transmitted disease associated with HPV infection. Other factors are involved such as immunosuppression and other sexually transmitted infections. Although surgical excision of the tumor seems to be the optimal therapeutic strategy. Early recognition and treatment will provide a good outcome.
